# Counter-Stereotypes and Feminism Promote Leadership Aspirations in Highly Identified Women

**DOI:** 10.3389/fpsyg.2017.00883

**Published:** 2017-06-02

**Authors:** Carola Leicht, Małgorzata A. Gocłowska, Jolien A. Van Breen, Soledad de Lemus, Georgina Randsley de Moura

**Affiliations:** ^1^Kent Business School, University of KentCanterbury, United Kingdom; ^2^Department of Work and Organizational Psychology, University of AmsterdamAmsterdam, Netherlands; ^3^Department of Psychology, University of GroningenGroningen, Netherlands; ^4^Department of Psychology, University of GranadaGranada, Spain; ^5^School of Psychology, University of KentCanterbury, United Kingdom

**Keywords:** women, gender identity, gender stereotypes, feminism, leadership

## Abstract

Although women who highly identify with other women are more susceptible to stereotype threat effects, women's identification might associate with greater leadership aspirations contingent on (1) counter-stereotype salience and (2) feminist identification. When gender counter-stereotypes are salient, women's identification should associate with greater leadership aspiration regardless of feminism, while when gender stereotypes are salient, women's identification would predict greater leadership aspirations contingent on a high level of feminist identification. In our study US-based women (*N* = 208) attended to gender stereotypic (vs. counter-stereotypic) content. We measured identification with women and identification with feminism, and, following the manipulation, leadership aspirations in an imagined work scenario. The interaction between identification with women, identification with feminism, and attention to stereotypes (vs. counter-stereotypes) significantly predicted leadership aspirations. In the counter-stereotypic condition women's identification associated with greater leadership aspirations regardless of feminist identification. In the stereotypic condition women's identification predicted leadership aspirations only at high levels of feminist identification. We conclude that salient counter-stereotypes and a strong identification with feminism may help high women identifiers increase their leadership aspirations.

## Introduction

Recent years have seen a proliferation of campaigns promoting gender equality. In 2005, the web designer Gretchen Cawthon launched a website called “Girls Can't What?”. The website sells merchandise featuring women in counter-stereotypic professions: e.g., women construction workers, women firefighters, and women scientists. In another campaign, in 2011, the gender studies department at the Massachusetts Institute of Technology promoted women's greater identification with feminism. As part of this campaign, members of the university received bags and badges promoting feminism, and were photographed holding signs saying, “This is what a feminist looks like”^1^. Events like these highlight an increasing recognition that gender disparities are detrimental to societies and economies, and suggest approaches that can be taken to address inequality. Our paper investigates two of these approaches: promoting gender counter-stereotypes, and increasing women's identification with feminism, and looks at the consequences they may have to women's leadership aspirations.

Studies suggest that campaigns stressing feminism and counter-stereotypes may help women to resist gender inequality, and that this is most likely in high women identifiers. Generally speaking, those who identify strongly with a group are most likely to protest against group-based inequality (Abrams and Randsley de Moura, 2002; Van Zomeren et al., 2008). However, as research shows, this is not always the case for gender. In fact past studies have found that high levels of gender identification associate with a greater susceptibility to threatening stereotypes, increasing the need for interventions targeting high women identifiers in particular. This suggests that additional conditions need to be met, to achieve high women identifiers' greater empowerment. While increased identification (e.g., with women) makes identity-related issues more relevant to the individual (Kaiser and Hagiwara, 2011), and can generally energize and motivate action (Van Knippenberg, 2000; Ellemers et al., 2004), the exact direction of those effects may depend on several moderators. For instance research on group identification has shown that its effects are contingent on salient identity cues (James and Greenberg, 1989; Van Knippenberg, 2000), and on specific identity *content* (Becker and Wagner, 2009) that may lead individuals to perceive certain actions (e.g., in this case, greater leadership aspirations) as beneficial for oneself or the in-group (Van Knippenberg, 2000). In consequence, the energizing effect of group identification on women's empowerment should depend on the salience of gender counter-stereotypic (vs. stereotypic) cues (Hoyt and Murphy, 2016), and on the presence of a politicized identification, like feminism, which sees resistance to stereotypic content as beneficial (Van Breen et al., in review). Based on this premise we asked whether counter-stereotype (vs. stereotype) salience, and feminist identification can moderate the relationship between women's identification and their leadership aspirations.

### Counter-stereotypes and leadership aspirations

Because leadership is typically associated with agency and masculinity (Sczesny and Kühnen, 2004; Hogue and Lord, 2007) and womanhood with nurturance and warmth (Eagly et al., 1992; Eagly and Karau, 2002), prevalent stereotypes create psychological barriers to women's participation in leadership (Eagly and Johnson, 1990; Rudman and Glick, 1999). Moreover, a heuristic tendency to prefer leaders that are perceived as embodying the ingroup's image (Hogg, 2001) makes it difficult for women to rise to leadership positions in male dominated domains. As such, stereotypes represent a considerable barrier to women's leadership aspirations. Indeed, past research has shown that exposing women participants to gender stereotypes can dampen their leadership aspirations (Davies et al., 2005). Likewise, under stereotype salience women report lower perceived performance, diminished leadership aspirations, diminished feeling of belonging, greater perceived task difficulty, and feelings of inferiority on leadership task (Davies et al., 2005; Cheryan et al., 2012, 2013). Additionally, when gender stereotypes are salient women may fear and/or experience backlash when they display leadership behavior (Eagly and Carli, 2007; Rudman and Fairchild, 2007; Amanatullah and Morris, 2010; Williams and Tiedens, 2015), leading them to avoid leadership roles to an ever greater extent. Conversely, increasing the salience of *counter*-stereotypes can maintain and even increase leadership aspirations in women (provided that these counter-stereotypes do not evoke threatening upward comparisons; Dasgupta and Asgari, 2004; Rudman and Phelan, 2010; Hoyt and Simon, 2011), and reduce the tendency to focus on in-group similarity (e.g., Leicht et al., 2014). One reason for these effects may be that counter-stereotypic content signals that it is safe, possible, and perhaps even desirable for women to behave in gender counter-stereotypic ways.

Taken together, when counter-stereotypes are salient, high identification with gender could energize women and increase their leadership aspirations. But women's behavior is not merely a function of group identification and of stereotype (vs. counter-stereotype) salience. Other factors are known to help women break out of stereotypic roles, and overcome disadvantage. This research suggests that women's reactions to gender issues (e.g., gender stereotypes) are determined not only by gender identification, but also by identification with feminism (Van Breen et al., in review, Van Breen et al., manuscript in preparation).

### Feminist identification and stereotype resistance

Gender constitutes one of people's most salient group identities, giving individuals a sense of belonging, and providing social roles and norms to live by (Tajfel, 1974; Banaji et al., 1993; Abrams and Hogg, 2004). But whilst much research on gender and performance focuses on the extent of women's gender identification (Kaiser and Hagiwara, 2011), how women construe gender identity is rarely considered. Becker and Wagner (2009) (also see Condor, 1986) proposed that when studying social identities researchers should consider not only the strength of people's identification, but also specific identity content (Becker and Wagner, 2009). This issue may be particularly important in the case of gender identification, because as research shows, feeling strong ties with other women does not preclude the perpetuation of gender inequality (Klandermans, 2014).

In an attempt to better understand how women construe their identity in relation to gender, recent research distinguished women's identification with other women from a second, orthogonal factor, namely women's identification with feminism. This research demonstrated that identification with feminism and with women constitute distinct and unique social identities, and two independent psychological constructs (Roy et al., 2007; Van Breen et al., in review). Extending those findings, we propose that in addition to women's gender identification, also identification with feminism may shape how women react to gender stereotypes. One possibility in particular, is that when stereotypic content is salient, high levels of feminist identification will direct high women identifiers' motivation toward actions that are considered beneficial for the group—such as stereotype resistance (Van Knippenberg, 2000; de Lemus et al., 2013; Van Breen et al., in review). This effect could be similar to what is observed in empowered women. For example, women entrepreneurs (Gupta et al., 2008) and women in high power positions (Hoyt and Blascovich, 2007) have been found to perform better than men when gender divisions are salient. A similar resistance effect may also be observed in high feminist identifiers. In other words, it is possible that, when stereotypes are salient and feminist identification is high, women's identification may associate with greater leadership aspirations.

In sum, our literature review suggests that women's identification should associate with greater leadership aspirations through two different “routes” (1) under conditions of counter-stereotype salience (in all research participants) and (2) under conditions of stereotype salience, but only provided that feminist identification is also high.

### This research

As highlighted, gender stereotypes (vs. counter-stereotypes) and feminist identification could moderate the link between women's identification and leadership aspirations. To test this idea we looked at the association between women's identification with other women and their leadership aspirations under conditions of stereotype (vs. counter-stereotype) salience, and in high (vs. low) feminist identifiers. We predicted that when gender counter-stereotypes are salient, women's identification should associate with greater leadership aspiration regardless of feminism, while when gender stereotypes are salient, women's identification would predict greater leadership aspirations contingent on a high level of feminist identification. We chose leadership aspirations as our dependent variable because leadership is counter-stereotypic for women and persistence in this domain signals women's greater resistance to stereotypic content. For exploratory reasons, we also measured effects to participants' fear of backlash.

## Pre-test study

Before running the main study we developed and pre-tested a novel manipulation of stereotype (vs. counter-stereotype) salience. We wanted to manipulate attention to stereotypes (vs. counter-stereotypes) without evoking threatening social comparisons (e.g., asking participants to think of a highly successful female CEO is potentially threatening, and may wipe out the effect of the counter-stereotype; Hoyt and Simon, 2011). To achieve this, we decided to present participants with neutral images of women and ask them to focus on how the women targets are portrayed in a stereotypic (vs. counter-stereotypic) manner. This type of manipulation, where the content of the image is kept constant across both conditions, has the additional benefit of being “clean” and confound-free. Typical manipulations of stereotype and counter-stereotype exposure ask participants to think of different targets, for instance Angela Merkel vs. Bill Clinton (Latu et al., 2013), or a “female mechanic” vs. a “male mechanic” (Gocłowska et al., 2014; Leicht et al., 2014), potentially confounding stereotype (vs. counter-stereotype) exposure with other features of the target (e.g., gender). Our new paradigm avoids these potential limitations, allowing us to assess the impact of stereotype (vs. counter-stereotype) salience and nothing else.

We pretested four images of women (available from authors upon request). Image A depicted a woman standing at a large window, image B a woman in front of a laptop holding a cell phone and a baby, image C a woman eating a salad in front of a computer, and imaged D a woman aircraft pilot with a military aircraft in the background. Next, we asked several questions about the target's stereotypicality and femininity.

The first goal of the pretest was to select (for the main study) two images that are most neutral in terms of stereotypicality and femininity. Using neutral imagery would ensure that the targets used in our manipulation are relevant to all research participants, and that they can be perceived as both stereotypic as counter-stereotypic, depending on instructions used. The second goal was to test the effectiveness of our new stereotype (vs. counter-stereotype) attention manipulation. Asking participants to describe the stereotypic (vs. counter-stereotypic) features of the (relatively neutral) female targets, should change participants' perceptions of those targets in line with the manipulation. Most likely, focusing on stereotypic (vs. counter-stereotypic) content should amplify ratings of stereotypicality and femininity. Competence, likability and attractiveness, emotional reactions and task difficulty could also potentially be affected, and were thus measured as well.

### Methods

US based MTurk workers (*N* = 259; 101 male, 157 female, 1 gender not specified) took part in an online survey (*M*_time_ = 12 min, $1 reward)^2^. Demographic questions indicated that 12% of participants were students, 13% were self-employed, 59% were full or part-time employed, 10% were homemakers, and 6% were jobseekers. We also inquired about gender distribution in the research participants' main organization (company, university etc.). According to participants' estimates, among “ordinary” workers with no managerial responsibilities 53% were female, among lower and middle management employees 48% were female, while among upper management employees 37% were female. Those numbers seem to confirm the general observation that women are underrepresented in upper management positions.

At the beginning of the study participants were allocated randomly to one condition in a 4 (Image: A vs. B vs. C vs. D) by 2 (Attention: counter-stereotypic vs. stereotypic) between-participants design. Within each condition we asked participants to describe the image they were presented with for at least 90 s. In the stereotypic condition we asked participants to describe the image focusing on its “*traditional and female gender stereotypic aspects”*, whilst in the counter-stereotypic condition we asked participants to focus on “*non-traditional and female gender counter-stereotypic aspects”*. After participants described the image, we asked about their perception of the task and of the stimuli.

All items were measured on a 7-point Likert-type scale (1, *not at all*; 7, *very much*). To measure target stereotypicality we used three items: *traditional, stereotypic* or *contrary to what society expects* (α = 0.73). Target femininity was measured across various domains (e.g., *behavior, posture, fashion;* α = 0.87). For exploratory purposes we asked participants to indicate *how attractive, how competent* and *how likeable* the target was (we treated those as separate variables), and to rate positive (e.g., happy, enthusiastic, α = 0.89) and negative (e.g., disappointed, anxious, α = 0.90) emotions evoked by the target. Finally, we also asked participants to indicate how easy or pleasant the task was (e.g., *How much did you enjoy describing the image*? *How easy it was to describe the image?*; 5-item measure, α = 0.85).

### Results

Table 1 represents mean ratings and pairwise comparisons of the four types of images. Images differed significantly in terms of stereotypicality [*F*_(3, 238)_ = 29.48, *p* < 0.001, η^2^ = 0.27] and femininity [*F*_(3, 238)_ = 25.76, *p* < 0.001, η^2^ = 0.25]. Pairwise comparisons (*post-hoc* LSD test) showed that image C (woman eating a salad) was perceived as overly stereotypic and feminine, while image D (woman pilot) was overly counter-stereotypic and relatively low on femininity. Based on those results we decided to only use Image A (woman in front of the window) and Image B (woman with baby and laptop) in the main study.

**Table 1:** Differences in the ratings of the four stimuli in Pretest Study and in Study 1.

		**Image type**							
		**Image A**		**Image B**		**Image C**		**Image D**	
		***M***	***SD***	***M***	***SD***	***M***	***SD***	***M***	***SD***
Pretest	Stereotypicality	3.92^a^	1.53	4.07^a^	1.31	4.58^c^	1.14	2.47^d^	1.03
	Femininity	4.90^a^	1.18	4.98^a^	1.05	5.50^c^	0.96	3.75^d^	1.25
	Attractiveness	6.07^a^	0.92	5.58^b^	1.08	6.12^a^	0.82	6.00^a^	0.94
	Likability	5.35^a^	1.27	5.73^b^	0.96	6.09^b^	0.95	5.67^b^	1.12
	Competence	5.60^a^	1.24	6.00^a^	0.98	5.95^a^	0.98	5.91^a^	1.02
	Positive emotions	3.53^a^	1.47	3.50^a^	1.55	3.67^a^	1.53	3.94^a^	1.52
	Negative emotions	1.65^a^	1.12	1.81^a^	1.18	1.58^a^	1.12	1.56^a^	1.03
	Task perception	4.91^a^	1.37	5.17^a^	1.23	5.11^a^	1.17	5.34^a^	1.24
Study 1	Stereotypicality	3.93^a^	1.26	4.12^a^	1.46	–	–	–	–
	Femininity	5.15^a^	1.15	5.02^a^	1.03	–	–	–	–
	Attractiveness	6.18^a^	0.82	5.36^b^	1.23	–	–	–	–
	Likability	5.86^a^	1.07	5.71^a^	1.07	–	–	–	–
	Competence	6.13^a^	1.03	6.19^a^	0.92	–	–	–	–
	Positive emotions	3.48^a^	1.56	3.53^a^	1.59	–	–	–	–
	Negative emotions	1.47^a^	0.83	1.68^a^	1.09	–	–	–	–
	Task perception	5.10^a^	1.37	5.63^b^	1.02	–	–	–	–

*Means with a different superscript are significantly different from one another at p < 0.05. Image A represented a woman looking out of a window, Image B a woman with a baby, mobile phone, and a laptop, Image C a woman eating lunch in front of a computer, and image D a woman pilot in front of a military aircraft. Images can be obtained from authors' upon request*.

Table 2 represents mean image ratings in the counter-stereotypic and stereotypic condition. As we hoped for, images in the stereotypic (vs. counter-stereotypic) condition were perceived as more stereotypic [*F*_(1, 240)_ = 21.78, *p* < 0.001, η^2^ = 0.08] and more feminine [*F*_(1, 240)_ = 12.43, *p* < 0.001, η^2^ = 0.05]. Additional exploratory analyses demonstrated that the stereotypic condition decreased ratings of competence [*F*_(1, 240)_ = 8.07, *p* = 0.005, η^2^ = 0.03], and positive emotions evoked by the target [*F*_(1, 240)_ = 7.93, *p* = 0.005, η^2^ = 0.03]. The manipulation had no effect on ratings of attractiveness or likability or on negative emotions and the perceived task difficulty. Overall, this set of findings suggests that our manipulation changed perceptions of targets in line with gender stereotypic (vs. counter-stereotypic) content. In addition, targets in the counter-stereotypic condition evoked greater (positive and negative) emotion and were seen as more competent.

**Table 2:** Differences in ratings between the counter-stereotypic and stereotypic condition in the Pretest Study and in Study 1.

		**Condition**			
		**Counter-stereotypic**		**Stereotypic**	
		***M***	***SD***	***M***	***SD***
Pretest	Stereotypicality	3.38^a^	1.35	4.23^b^	1.49
	Femininity	4.54^a^	1.31	5.10^b^	1.17
	Attractiveness	5.89^a^	0.96	6.00^a^	0.96
	Likability	5.81^a^	1.02	5.62^a^	1.18
	Competence	6.06^a^	0.93	5.68^b^	1.15
	Positive emotions	3.92^a^	1.53	3.38^b^	1.47
	Negative emotions	1.69^a^	1.19	1.61^a^	1.04
	Task perception	5.12^a^	1.23	5.13^a^	1.28
Study 1	Stereotypicality	3.43^a^	1.27	4.62^b^	1.17
	Femininity	4.81^a^	1.18	5.37^b^	0.92
	Attractiveness	5.80^a^	1.16	5.76^a^	1.07
	Likability	5.85^a^	1.10	5.73^a^	1.04
	Competence	6.35^a^	0.82	5.96^b^	1.08
	Positive emotions	3.79^a^	1.56	3.21^b^	1.53
	Negative emotions	1.53^a^	0.95	1.62^a^	0.99
	Task perception	5.31^a^	1.31	5.40^a^	1.16

*Means with a different superscript are significantly different from one another at p < 0.05. Pretest ratings represent aggregated ratings of four images (A, B, C, and D) while Study 1 ratings represent aggregated ratings of two images selected from the pretest (A, B)*.

## Main study

Having pre-tested our manipulation and selected the two most neutral images, we set out to run the main study. We asked participants to attend to stereotypic (or counter-stereotypic) features of the selected stimuli, and measured leadership aspirations and identification with women and with feminism. Our goal was to test whether the three-way interaction of women's identification, feminist identification and stereotype salience would predict women's leadership aspirations.

### Methods

#### Participants, design, and procedure

Using MTurk, 238^3^ female US based workers (*M*_age_ = 36.06, *SD* = 12.23) were recruited to take part in an online study on “image perception and description,” and were allocated randomly to the stereotypic or counter-stereotypic attention condition. The questionnaire took, on average, 31 min to complete, and participation was rewarded with $1.5.Participants' professional status was the following: 9% students, 12% self-employed, 55% full or part-time employed, 16% homemaker, 8% jobseeker. Also in this sample, gender distribution was perceived as being unequal in the highest ranks in the organization. Among “ordinary” workers with no managerial responsibilities 51% were thought to be female, among lower and middle management employees 49% were female, while among upper management employees 38% were reported to be female.

After completing informed consent participants were presented with one of our two selected images. We used and counter-balanced (between-participants) two images to ensure greater external validity and generalizability of our findings^4^. As in the pre-test study we asked participants to identify image elements portraying the woman in a stereotypic/traditional or non-stereotypic/non-traditional way. Participants had to spend at least 90 s on this task (more time was allowed as well). We then measured leadership aspirations. We instructed research participants to imagine a workplace scenario and indicate to what extent they would like to and would feel comfortable taking a leadership role in that situations. For exploratory purposes we also measured participants' fear of backlash. Both variables were measured in a counterbalanced way, to account for order effects.

Following our manipulation and the workplace scenario, we measured several individual difference moderators and covariates. This part of the questionnaire inquired about participants' gender, their identification with women and with feminism, and several individual difference measures related to feminism. To account for individual differences in people's beliefs about gender (Morton et al., 2009; Napier et al., 2010; Okimoto and Brescoll, 2010), we asked participants to fill in measures of gender essentialism, gender system justification, benevolent sexism and liberal feminist attitudes (Glick and Fiske, 1996; Jost and Kay, 2005; Napier et al., 2010; Connelly and Heesacker, 2012; Brescoll et al., 2013; de Lemus et al., 2015). We wanted to use those measures as covariates, to see whether the effects uncovered are *specifically* due to feminist identification, rather than merely feminist beliefs. If effects of feminist identification (and it's interaction with other variables) held regardless of other gender related constructs, this would indicate that our effects are specific to feminist *identification*.

Because this part of the questionnaire came after the manipulation, we took precautions to ensure that participants report their stable beliefs and level of identification. Namely, before measuring identification and gender-based beliefs we instructed participants (several times) to “express to what extent you generally agree with the statements below” and to provide answers based on “your attitudes, beliefs and experiences most of the time in your everyday life.” Furthermore, we also inspected the data to see whether the manipulation had any effects on the individual differences: none effects were uncovered (*p*s > 0.52). This finding is consistent with that of other research measuring identification *after* the manipulation (Jimenez-Moya et al., 2015).

Finally, we reminded participants of the stereotype (vs. counter-stereotype) salience manipulation, and asked them to complete the same measures as in the pre-test, except that this time we treated stereotypicality and femininity as manipulation checks.

#### Dependent variables

All measures in the study were captured using a Likert-type scale with 1 indicating “not at all” and 7 indicating “very much.” For correlations of all the measures in this study see Table 3.

**Table 3:** Bivariate correlations between dependent variables, moderators, covariates, and manipulation checks.

	**Variables**	**1**	**2**	**3**	**4**	**5**	**6**	**7**	**8**	**9**	**10**	**11**	**12**	**13**	**14**	**15**
Dependent variables	(1) Leadership aspirations															
	(2) Fear of backlash	−0.42^**^														
Moderators	(3) Women identification	0.19^**^	−0.15^*^													
	(4) Feminist identification	−0.03	0.18^*^	0.01												
Covariates	(5) LFAIS	0.02	0.01	0.03	0.57^**^											
	(6) GSJ	0.16^*^	−0.19^**^	0.16^*^	−0.43^**^	−0.58^**^										
	(7) Gender essentialism	−0.02	0.12	0.13	−0.30^**^	−0.52^**^	0.42^**^									
	(8) Benevolent sexism	0.09	−0.10	0.23^**^	−0.15^*^	−0.14^*^	0.37^**^	0.47^**^								
Manipulation checks	(9) Stereotypicality	0.12	0.07	0.07	0.11	0.07	−0.04	−0.02	−0.04							
	(10) Femininity	0.06	0.01	0.06	0.10	0.12	0.02	0.04	0.15^*^	0.23^**^						
	(11) Attractiveness	0.11	0.00	0.22^**^	0.15^*^	0.12	0.09	−0.05	0.06	−0.15^*^	0.36^**^					
	(12) Likeability	0.21^**^	−0.13	0.20^**^	0.07	0.12	0.09	−0.08	0.10	−0.10	0.28^**^	0.66^**^				
	(13) Competence	0.05	−0.07	0.17^*^	0.04	0.11	0.03	−0.06	0.08	−0.20^**^	0.09	0.48^**^	0.61^**^			
	(14) Positive emotions	0.19^**^	−0.11	0.17^*^	0.02	−0.03	0.22^**^	0.01	0.22^**^	−0.17^*^	−0.06	0.28^**^	0.36^**^	0.36^**^		
	(15) Negative emotions	−0.06	0.23^**^	0.02	0.11	0.09	−0.15^*^	0.11	0.04	0.21^**^	0.12	−0.09	−0.11	−0.15^*^	−0.05	
	(16) Task perception	0.17^*^	−0.12	0.12	0.04	0.07	0.16^*^	0.02	0.25^**^	0.06	0.24^**^	0.20^**^	0.38^**^	0.30^**^	0.36^**^	0.00

** p < 0.001;

* *p < 0.01; GSJ, Gender System Justification; LFAIS, Liberal Feminist Attitudes and Ideology Scale*.

We embedded the leadership aspirations and fear of backlash measures in an imagined work scenario. First, we asked participants to think of a place where they frequently are, or have been involved in group projects. We asked them to focus on a place that is most current and relevant to what they do, and to write the name of this place down in the box provided. Common responses given by participants were “work,” “office,” “school,” “university,” “college,” or “church”. On the next page we referred to this location, and asked participants to imagine the following situation:

“On a typical day at [location name provided by research participant] you were selected to be part of a new project team, responsible for completing a specific task. You know who your team members are, and you know that all of you have similar expertise and experience on this type of project.”

We then asked participants to answer several questions about their leadership aspirations and fear of backlash in the context of this event. Leadership aspirations were measured by asking participant to indicate on 13 statements whether they would like to take on the leadership role within this group (e.g., “I would like to be selected as a leader for this task.”). These statements were mostly self-created, with a few borrowed from previous research (α = 0.95) (Hoyt and Simon, 2011). For an overview of the items see **Appendix** at the end of this paper. Fear of backlash was also assessed within this imaginary work workplace scenario, however, rather than assessing whether participants would like to take on the leadership role, we asked about participants' level of (dis)comfort in leadership positions. Using four items borrowed from previous research (Moss-Racusin and Rudman, 2010) we asked each participant how (un)comfortable she would feel being appointed as a leader (α = 80; e.g., “I would be concerned that I might be disliked.”). For fear of backlash, the instruction additionally stressed that the research participant was the group member with the highest expertise for the task.

#### Moderators

Identification with women and with feminists was measured using two parallel sets of items. Three items measured participants' identification with women, (e.g., “Being a woman is an important part of who I am”; α = 0.82), and three similar items were used to measure identification with feminism, replacing the word “woman” with “feminist,” α = 0.99 (de Lemus et al., 2015).

#### Covariates

Gender System Justification (GSJ), Gender Essentialism (GE), Benevolent Sexism (BS) were measured using well-established measures with good reliability, α_GSJ_ = 0.72, α_BS_ = 0.91, α_GE_ = 0.83. (Glick and Fiske, 1996; Jost and Kay, 2005; Okimoto and Brescoll, 2010). In order to distinguish between identification with feminism, and attitudes toward feminism, we also asked participants to fill in the 14 item Liberal Feminist Attitudes and Ideology Scale (LFAIS; α = 0.89). Here participants were asked to indicate the extent to which they agreed with statements such as “A woman should have the same job opportunities as a man.” (Levonian Morgan, 1996).

#### Manipulation checks

Using items identical to those in the pretest we measured stereotypicality (α = 0.58), femininity (α = 0.85), competence (one item), likeability (one item), and attractiveness (one item) of the model as well as positive (α = 0.91) and negative emotions (α = 0.91), and task difficulty (α = 0.85). For correlations between all measures see Table 3.

## Results

### Collinearity check

#### Gender identity, feminist identity, and feminist attitudes

Our hypotheses were tested with moderated regression analysis in PROCESS macro (Hayes, 2013). Before running these analyses, we conducted a collinearity check on our two identity predictors and a content related co-variate of feminist attitudes. This would ensure that gender identification, feminist identification, and feminist attitudes are distinct constructs. First, we conducted a Factor Analysis with all items from all scales included in the analysis. With a varimax rotation, the analysis revealed a four factor structure explaining 70.45% of variance. The first two factors were formed out of the liberal feminist attitudes scale, with all positive phrased items loading on Factor 1 and all negative phrased items loading on Factor 2. The third factor consisted out of the three gender identification items, whereas the fourth factor consisted out of the three feminist identification items. Correlation analyses (see Table 3) additionally revealed that the liberal feminist attitude scale was related to feminist identification, but not with women's identification or leadership aspirations.

In summary, all three scales formed distinct factors that corresponded with our variables, gender identification, feminist identification, and liberal feminist attitudes, none of the items cross loaded, and correlations were non-significant between our two predictor variables, gender identification and feminist identification. This confirms that feminist identification and women's identification are distinct factors, and that that including these variables as predictors and covariates in a regression analysis is not problematic (Antonakis et al., 2010).

### Manipulation check

Results of the manipulation check were very similar to what we found in the pretest. Table 1 (bottom panel) shows mean ratings across the two types of images used. As in the pretest, image A and image B did not differ significantly in terms of stereotypicality or femininity. More importantly to our argument, mean image ratings in the counter-stereotypic and stereotypic condition (Table 2) demonstrated that the manipulation worked as intended. Namely, images in the stereotypic condition were perceived as more stereotypic [*F*_(1, 206)_ = 49.06, *p* < 0.001, η^2^ = 0.19] and more feminine [*F*_(1, 206)_ = 14.96, *p* < 0.001, η^2^ = 0.07]. The targets in the stereotypic condition were also seen as less competent [*F*_(1, 206)_ = 8.64, *p* = 0.004, η^2^ = 0.04], and elicited less positive emotions [*F*_(1, 206)_ = 7.26, *p* = 0.008, η^2^ = 0.03]. Thus, as intended, our manipulation changed perceptions of targets in line with gender stereotypic (vs. counter-stereotypic) content.

### Hypothesis test

The main goal of our study was to examine under what conditions women's gender identification links with greater leadership aspirations. We tested this idea using the PROCESS macro Model 3 (Preacher and Hayes, 2008). We entered leadership aspirations as the dependent variable, and gender identification, feminist identification and attention (0 = attention to counter-stereotypic information, 1 = attention to stereotypic information) as predictor variables.

#### Leadership aspirations

**Figure 1:**
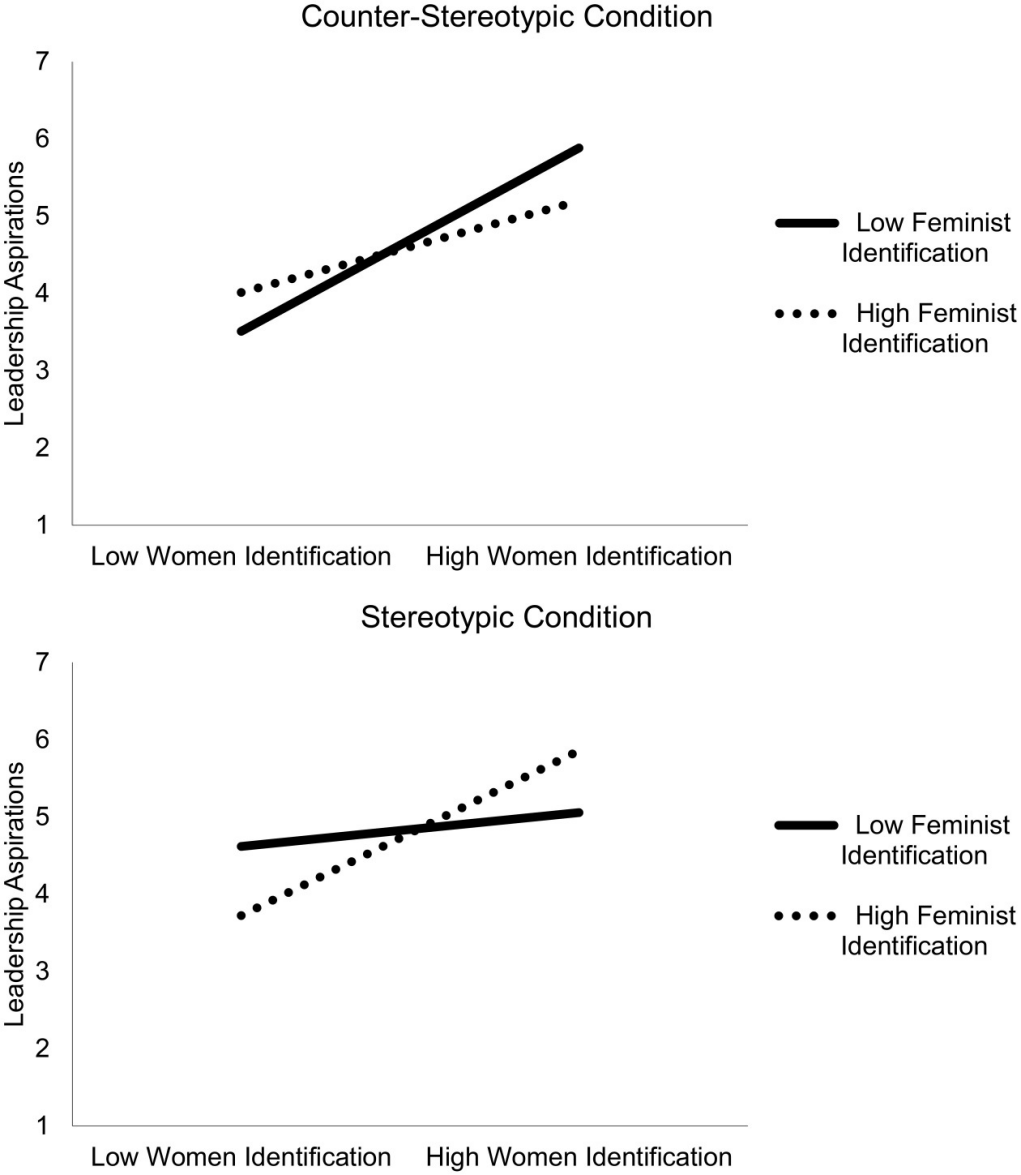
Leadership aspirations as a function of feminist and women identification in the counter-stereotypic condition **(upper)** and in the stereotypic condition **(lower)**.

The analysis revealed a significant main effect for gender identification, *B* = 0.32, *SE* = 0.13, *t*_(200)_ = 2.50, *p* = 0.013 95*%CI* [0.07;0.57], showing that identification with gender was positively associated with leadership aspirations (also see Table 4, left panel). There were no main effects of feminist identification and attention on leadership aspirations (all *p's* > 0.46). The effect of gender identification was qualified by a significant three-way interaction between gender identification, feminist identification and attention, *B* = 0.28, *SE* = 0.11, *t*_(200)_ = 2.48, *p* = 0.01 95%*CI* [0.06;0.51], which significantly increased the total amount of variance explained, *R*^2^ = 0.07, *p* = 0.03; *R*^2^—*change* = 0.03, *p* = 0.01. To break down the interaction term, we split the sample by condition and entered feminist identification and women's identification as the independent variables, and leadership aspirations as the dependent variable (see Figure 1).

**Table 4:** Results of the key regression analyses with leadership aspirations entered as the dependent variable.

	**Main analysis**					**Sensitivity analysis (adding control variables)**				
				**95%CI**					**95%CI**	
	***B***	***t***	***p***	**Lower**	**Upper**	***B***	***t***	***p***	**Lower**	**Upper**
Fem.Id.	−0.03	−0.58	0.57	−0.11	0.06	−0.04	−0.70	0.48	−0.15	0.07
Wom.Id.	**0.32**	**2.50**	**0.01**	**0.07**	**0.57**	0.22	1.69	0.09	−0.04	0.49
Wom.Id. × Fem.Id.	0.03	0.58	0.56	−0.08	0.15	0.02	0.39	0.69	−0.09	0.13
Condition	0.15	0.73	0.46	−0.25	0.54	0.26	1.28	0.20	−0.14	0.65
Wom.Id. × Condition	−0.15	−0.59	0.55	−0.66	0.35	−0.01	−0.03	0.97	−0.52	0.50
Fem.Id. × Condition	0.00	0.05	0.96	−0.17	0.18	−0.01	0.06	0.95	−0.18	0.17
Wom.Id. × Fem.Id. × Condition	**0.28**	**2.49**	**0.01**	**0.06**	**0.51**	**0.33**	**2.86**	**0.00**	**0.10**	**0.55**
GSJ						0.31	1.78	0.08	−0.03	0.66
Gender essentiallism						−0.04	−0.33	0.74	−0.26	0.18
Benevolent sexism						−0.02	−0.22	0.83	−0.107	0.14
LFAIS						0.18	1.29	0.20	−0.10	0.46
Positive emotions						**0.16**	**2.27**	**0.02**	**0.02**	**0.29**
Negative emotions						−0.09	−0.86	0.39	−0.30	0.12

*Sensitivity Analysis represent the key finding when controlling for gender relevant variables. Significant predictors are marked in bold. Fem.Id., Feminist Identification; Wom.Id., Identification with Women; Wom.Id. x Fem.Id., Interaction term of identification with women and with feminists; Condition: counter-stereotypic coded as “0” and stereotypic coded as “1”; Wom.Id. x Condition, Interaction term of identification with women and of condition; Fem.Id. x Condition, Interaction term of identification with feminism and of condition; Wom.Id. x Fem.Id. x Condition, three way interaction term of the two identifications and the condition; GSJ, Gender System Justification; LFAIS, Liberal Feminist Attitudes and Ideology Scale*.

In the counter-stereotypic condition there was a significant main effect of women's identification: *B* = 0.42, *SE* = 0.18, *t*_(101)_ = 2.33, *p* = 0.02 95%*CI* [0.06;0.77], but feminist identification and the interaction terms did not emerge as significant predictors (*p*s > 0.22). Notably, inspecting the standardized regression coefficients revealed that the effect of women's identification on leadership aspirations was higher in the counter-stereotypic condition (β = 0.239), than in the overall sample (β = 0.186). In other words, women who were highly identified with other women (vs. those who were not) showed increased leadership aspirations after attending to counter-stereotypic information.

In the stereotypic condition the significant three way interaction was explained by a two way interaction between gender identification and feminist identification: *B* = 0.18, *SE* = 0.07, *t*_(99)_ = 2.34, *p* = 0.02, 95%*CI* [0.03;0.33]. When further broken down, this significant interaction in the stereotypic condition resulted in one significant slope. Gender identification was associated with higher leadership aspirations in the stereotypic condition, however this was only the case if identification with feminism was also high, *B* = 0.68, *SE* = 0.26, *t*_(99)_ = 2.66, *p* = 0.01, 95%*CI* [0.16;1.20]. Put differently, while under counter-stereotype salience women's identification associated with greater leadership aspirations regardless of feminism, under stereotype salience women's identification associated with greater leadership aspirations only when feminist identification was high.

To test for the robustness of those results, we re-run our analyses with Gender System Justification, Benevolent Sexism, Gender Essentialism, positive and negative emotions, and the Liberal Feminist Attitudes Scale as covariates (See Table 4, right panel). When controlling for these variables, the effect of the three-way interaction term of stereotype exposure and women and feminist identification was still significant: *B* = 0.33, *SE* = 0.11, *t*_(194)_ = 2.86, *p* < 0.01, 95%*CI* [0.10;0.55].

#### Fear of backlash

For exploratory purposes we conducted identical analyses with fear of backlash as a dependent variable. The analyses revealed a significant main effect of feminist identification on fear of backlash, *B* = 0.09, *SE* = 0.04, *t*_(200)_ = 2.45, *p* = 0.02, 95%*CI* [0.02;0.17], so that higher feminist identification was associated with greater fear of backlash. There was also a marginal negative effect of gender identification, *B* = −0.20, *SE* = 0.11, *t*_(200)_ = −1.90, *p* = 0.06, 95%*CI* [−0.42;0.011]. Attention to stereotypes was not significant, and there were no significant interaction effects (all *ps* > 0.26).

## General discussion

Counter-stereotypes and feminism are thought to inspire and motivate women to challenge gender inequality, and are often used in public campaigns and interventions. Could such means be helpful in raising the leadership aspirations of highly identified women? To answer this question we tested whether stereotype (vs. counter-stereotype) exposure and feminist identification moderate the effect of gender identification on women's leadership aspirations. A review of the literature suggested that high gender identification should associate with greater leadership aspirations (1) under conditions of counter-stereotype salience (in all research participants) and (2) under conditions of stereotype salience, conditional on greater levels of feminist identification. Those predictions were based on what we know about women's reactions to non-threatening counter-stereotypes (Hoyt and Simon, 2011; Latu et al., 2013), and based on the literature on stereotype resistance (de Lemus et al., 2015; Van Breen et al., in review). In line with these expectations we uncovered that stereotype (vs. counter-stereotype) salience and participant's identification with women and with feminism interacted to predict female participants' leadership aspirations. The three-way interaction term was further explained by a significant effect of women's identification (regardless of feminist identification) on leadership aspirations in the counter-stereotypic condition, and by a significant effect of women's identification on leadership aspirations in the stereotypic condition (but only in high feminist identifiers). These findings are in line with recent research showing that gender identification and feminist identification are orthogonal identity constructs, and that women's reactions to gender stereotypes and to counter-stereotypes depend the interaction of these two identities (de Lemus et al., 2015; Van Breen et al., in review). Our research extends those findings by showing that women's and feminist identification interact with stereotype salience to affect behavioral intentions such as leadership aspirations.

### Limitations and future directions

There are a number of limitations regarding the operationalization of theoretical constructs, as well as the generalizability of the research results. For example, leadership aspirations were measured using self-report, rather than actual leadership behavior. Agreeing with this criticism, we perceive the present set of findings as a preliminary step, rather than a definitive statement, about the role that identification and stereotypes (vs. counter-stereotypes) play in women's leadership aspirations. We advocate that in the future more research is needed to investigate whether the effects found in the present study carry over to women's performance on actual leadership tasks, such as tasks that require of women to lead on a team project, or to presenting a speech in front of their team members (Latu et al., 2013). Furthermore, more research on this topic could be conducted in organizational settings and in active women leaders. For example looking at whether in traditional organizations (where gender stereotypes abound) women who endorse progressive identification are more often promoted to leadership roles would garner more support for and extend the present set of findings. Although correlational in nature, this type of test would suggest that not only do high women high feminist identifiers have greater leadership aspirations, but that they also successfully realize these aspirations. This type of test would allow for a better understanding of the extent to which the processes investigated herein can also be found in “real-life” settings.

### Theoretical and practical implications

The current findings have several implications for theory and practice. First of all, they demonstrate, in line with other social identity research, that higher levels of identification with one's group can fuel individuals' motivation, but that the direction of those effects depends on variables related to identity salience and to one's perception of what is beneficial to oneself and the in-group. In line with this reasoning, our results show that the effect of gender identification apparent in our study is contingent on the salience of counter-stereotypic or stereotypic identity cues (James and Greenberg, 1989; Worchel et al., 1998; Van Knippenberg, 2000; Hoyt and Murphy, 2016), and on a politicized identification (feminism) that encourages resistance toward stereotypic norms (Van Breen et al., in review). Altogether these findings support the idea that counter-stereotypes and feminism can help increase women's aspirations, and that this is especially the case for women highly identified with their in-group.

Secondly, consistent with recent research on women's identification (Van Breen et al., in review), our study supports the idea that gender identification and feminist identification are unique constructs, and that they can shape women's reactions to stereotypic or counter-stereotypic information. Van Breen et al. (in review) proposed that whilst some women have a more traditional gender identification, and identify highly with women, but not with feminism (“traditional women”), others have a more progressive understanding of their gender identity, and identify highly with both women and with feminism (“dual identifiers”), only with feminism (“distinctive feminists”), or with none (“low identifiers”). Interpreting our results through this lens suggests that the distinction between gender and feminist identification is especially important in a salient gender stereotypic context. Namely, in this context we see that when stereotypes are salient, progressive women (who identify highly with women and with feminism) have greater leadership aspirations than distinctive identifiers (who identifying highly with feminism but not with women). This finding is very interesting as it opens up new interpretations on the role of group identification in stereotypic contexts. Stereotype threat literature has shown some pessimistic indications that highly identified women are at more risk from stereotype threat (Kaiser and Hagiwara, 2011). While we believe this to be true, we also think that gender identification can have beneficial effects for women—provided that it is supplemented with some kind of belief, or a second type of identity, that inoculates women against the debilitating effects of stereotype salience. The present set of findings suggests that when feminist identification is high, stereotypic content fuels leadership aspirations amongst those who are strongly identified with the group. We believe that this finding opens up a new and exciting possibility for stereotype threat research: that fostering a feminist identification can *inoculate* women against threatening stereotypic content. Moreover, the current set of findings may even suggest that feminist identification *promotes* leadership aspirations, as much as counter-stereotypes do. This finding emphasizes the importance of resistance to stereotypes as a motivated response to protect the interests of the group (de Lemus et al., 2015; Van Breen et al., in review).

At this point in the discussion an inquisitive reader may ask why, in the present study, women's identification was related to leadership aspirations (contingent on counter-stereotype salience and on feminism), but feminism was not. Since feminism is often associated with a greater readiness for collective action (Bliuc et al., 2007; Klandermans, 2014), and with the endorsement of women in gender counter-stereotypic roles, one might expect a link between women's feminist identification and their greater leadership aspirations. Our study, on the other hand, shows that feminist identifiers do endorse greater feminist beliefs (lower gender system justification, gender essentialism and benevolent sexism, and higher level of liberal feminist attitudes), but not greater leadership aspirations. In fact, as the correlations in Table 3 indicate, leadership aspirations are not correlated with any of the feminist beliefs or gender beliefs. The only correlation that emerges with gender content is that of a *positive* relation between leadership aspirations and gender system justification. In addition, the data suggests that high leadership aspirations associate with greater perceived liking of the task, greater amount of positive emotions evoked by our image stimuli, and with greater ratings of model likeability. This may suggest that women with greater leadership aspirations are more dominant, confident and comfortable leaders, and that they see things in a more positive light. Thus, greater leadership aspirations are not *directly* linked to feminist beliefs, and can even lead to support for greater gender inequality. This observation is consistent with the “Queen Bee” phenomenon, showing that female leaders can sometimes be perpetrators of discrimination (e.g., Derks et al., 2015), and our data seem to support this point. Thus, if anything, leadership aspirations probably reflect one's resilience in light of societally imposed norms or barriers, rather than a politicized motivation to challenge gender inequality.

Next to these more theoretical contributions, our research also has several practical implications. It highlights the benefits of recent endeavors of policy makers and practitioners to increase greater the salience of gender counter-stereotypic behavior and women's identification with feminism. While most research focuses on the role of feminism to collective action, hereby we extend the benefits of feminist identification to organizational outcomes, such as leadership aspirations. Overall, our findings indicate that having a more progressive gender identity, that combines identification with women and with feminism, could have positive effects women's career progression, by motivating them to increase engagement in gender counter-stereotypic domains such as leadership.

## Ethics statement

This study was carried out in accordance with the recommendations of the American Psychological Association, with written informed consent from all subjects. All subjects gave written informed consent in accordance with the Declaration of Helsinki. The protocol was approved by the ethics committee of the School of Psychology at the University of Kent.

## Author contributions

CL and MG made an equal contribution to this project: they designed and conducted the studies, analyzed the data, and wrote the paper. SdL, JVB, and GRdM contributed to the project conception and provided critical comments and revisions.

### Conflict of interest statement

The authors declare that the research was conducted in the absence of any commercial or financial relationships that could be construed as a potential conflict of interest.

## Footnotes

^1^For the website of the Girls Can't What campaign follow this link: https://www.girlscantwhat.com. For images from the MIT campaign, follow this link: https://www.flickr.com/photos/59603194@N02/sets/72157626631504524/

^2^During study completion 16 participants failed attention checks, and their data were excluded from further analysis, leaving a final sample of 242 participants (96 male, 145 female, 1 gender not specified; *M*age = 38.15, *SD* = 12.74).

^3^Thirty participants failed the two attention checks included in our experiment and were therefore excluded from our sample, leaving us with a final sample of 208 participants for analysis (*M*_age_ = 36.60, *SD* = 12.46). Conducting the analyses with the full dataset did not change the significance of the results.

^4^The study also included a third factor: guided (vs. unguided) focus. In the guided condition participants were asked to focus on specific aspects of the image while focusing on stereotypic or counter-stereotypic features. In the unguided condition no specific instructions were given. This factor is of little theoretical significance, and did not emerge as a significant predictor.

## Appendix

### Leadership aspirations items

A = Items from Hoyt and Simon (2011).

R = Reverse coded items.

1. I would like to be the leader for this task. (A).
2. I would like someone else to be the leader for this task. (R).
3. I would volunteer to lead on this task.
4. I would be happy leading on this task.
5. I would be excited about leading on this task.
6. I would feel anxious about leading on this task. (R).
7. I would hope that I would NOT be selected as a leader for this task. (A) (R).
8. I could motivate others to do their best on this task.
9. I would be a team member that has considerable influence within the team.
10. I would enjoy taking on the leadership role within this team.
11. I would find it very stressful to take on the leadership role within this team. (R)
12. I could help in making the team successful.
13. I would lead this task effectively.
